# Problem Characteristics and Dynamic Search Balance-Based Artificial Bee Colony for the Optimization of Two-Tiered WSN Lifetime with Relay Nodes Deployment

**DOI:** 10.3390/s22228916

**Published:** 2022-11-18

**Authors:** Wenjie Yu, Xiangmei Li, Zhi Zeng, Miao Luo

**Affiliations:** 1 School of Automation, Chengdu University of Information Technology, Chengdu 610225, China; 2 School of Cybersecurity, Chengdu University of Information Technology, Chengdu 610225, China; 3 School of Mechanical and Electrical Engineering, University of Electronic Science and Technology of China, Chengdu 611731, China

**Keywords:** two-tiered wireless sensor networks, relay node deployment, Artificial Bee Colony, lifetime optimization, Swarm Intelligence

## Abstract

Lifetime optimization is one of the key issues among the many challenges of wireless sensor networks. The introduction of a small number of high-performance relay nodes can effectively improve the quality of the network services. However, how to deploy these nodes reasonably to fully enhance the network lifetime becomes a very difficult problem. In this study, a modified and enhanced Artificial Bee Colony is proposed to maximize the lifetime of a two-tiered wireless sensor network by optimal deployment of relay nodes. First, the dimension of the problem is introduced into the candidate search equation and the local search is adjusted according to the fitness of the problem and number of iterations, which helps to balance the exploration and exploitation ability of the algorithm. Second, in order to prevent the algorithm from falling into local convergence, a dynamic search balance strategy is proposed instead of the scout bee phase in the original Artificial Bee Colony. Then, a feasible solution formation method is proposed to ensure that the relay nodes can form the upper-layer backbone of the network. Finally, we employ this algorithm on a test dataset obtained from the literature. The simulation results show that the proposed algorithm for two-tiered wireless sensor network lifetime optimization can obtain higher and stable average network lifetime and more reasonable relay node deployment compared to other classical and state-of-the-art algorithms, verifying the competitive performance of the proposed algorithm.

## 1. Introduction

A wireless sensor network (WSN) is a multi-hop self-organizing information perception and data collection system. It can obtain detailed and accurate data in a variety of environments and pass the information between people and the objective world [1,2]. Among the many challenges in WSN applications, one of the most basic problems is how to deploy sensor nodes (SNs) reasonably in order to meet the service quality of the network.

In a WSN composed of ordinary nodes, the energy of the nodes is limited [3] and the communication burden of certain nodes is heavy, which easily causes the network to stop working prematurely [4]. To tackle this problem, a large number of redundant SNs generally need to be deployed in the network; however, this causes a lot of additional costs. The alternative solution is to reasonably deploy a small number of relay nodes (RNs) with high energy in the traditional WSN [5]; that is, the accurate calculation the location of RNs can help to replace the large number of redundant SNs deployed. Based on their deployment, related research can be divided into the categories of single-tiered and two-tiered WSN RN deployments [4]. All devices deployed in single-tiered WSN (ST-WSN) follow the same multi-hop routing rules except for the sink node. In the deployment of two-tiered WSN (TT-WSN), ordinary nodes only send data to RNs in one hop, and RNs only send information to sink nodes or other RNs. Finally, RNs send information to the sink.

Due to the high cost of RNs, scholars are greatly concerned with how to deploy the fewest RNs in a network. However, when there are a fixed number of RNs, another problem that cannot be ignored is how to deploy them to form the optimal upper-layer skeleton of the network in order to optimize its lifetime. This problem is NP-Hard [6], and must be solved with non-traditional methods.

Both RNs and SNs deployed in an ST-WSN forward the received packets. The deployment of a minimum number of RNs has been studied in a series of fixed candidate locations in a WSN with guaranteed network connectivity [7]. As an extension of this study, Misra et al. [8] investigated how to deploy a minimum number of RNs in an energy harvesting WSN while ensuring the connectivity and lifetime of the network. The candidate locations with energy harvesting potential were predetermined. Perez et al. [9] used a multi-objective algorithm to optimize the energy consumption and the number of routes for a single-tiered WSN. Nigam et al. [10] proposed a branch-and-cut algorithm to deploy a minimum number of RNs in certain candidate locations in an ST-WSN to ensure that the duration of sensor node and base station communication satisfies a predetermined delay bound. Truong et al. [11] proposed a multi-objective network repair algorithm to restore the connectivity of WSNs in unknown regions. Ozkan and Ermis [12] studied the problem of how to ensure network connectivity by deploying RNs in an ST-WSN. They transformed the RN deployment problem into a mixed integer programming model using genetic algorithms and simulated annealing algorithms to find the minimum number of RNs to be deployed and their reasonable locations. Lanza-Gutierrez and Gomez-Pulido [4] investigated how to deploy RNs in a ST-WSN based on a meta-heuristic algorithm. Their aims was to optimize the average energy consumption of the network as well as its average sensing area.

Compared to ST-WSNs, TT-WSNs are more balanced in energy consumption for SNs, although they are more complex in network topology formation. A genetic algorithm-based clustering method was proposed to optimize the lifetime of a TT-WSN with different delays in [13]. In their study, a multi-objective and top level GA was applied to obtain clustering schemes to optimize the network lifetime for different delay values. The low level GA is used in each cluster in order to find the most efficient topology for the transmission of data from sensor nodes to the cluster head. Azharuddin and Jana [14] employed a liner programming formulation for the TT-WSN deployment problem and then presented a genetic algorithm based meta-heuristic algorithm to minimize the number of RNs and maximize the connectivity of the network. Chen et al. [15] transformed the RN deployment problem into a minimum geometric disk coverage problem and determined that the problem is NP-complete. Then, they proposed a linear time approximation algorithm to solve it, in which the covering disks used the regular hexagon tessellation of the plane with bounded area. Hashim et al. [16] proposed an Artificial Bee Colony algorithm-based deployment method to extend the lifetime of a network by optimizing the parameters of the network and limiting the total number of RNs. Unlike their approach, the RNs in the present work are closer to SNs with limited energy. Yang et al. [17] used a heuristic algorithm to deploy the minimum number of RNs in a TT-WSN under the requirements of connectivity and lifetime satisfaction. To meet the survivability requirement, they studied the 2-connected double-cover problem where each sensor node was covered by two base stations or relay nodes, and the relay nodes formed a 2-connected network with the base stations. Ma et al. [18] used a tree-based connecting algorithm heuristic to optimize the network delay of TT-WSNs. They deployed RNs at a subset of predetermined deployment locations such that each sensor was covered by at least one RN, while ensuring the obedience of delay constraints. From the above studies, RNs are often deployed at certain discrete candidate locations; the energy of RN is limited, and there is a direct communication between each SN and RN. However, in practical applications the deployment space of RNs is often continuous, and RNs can be kept infinitely energetic by means such as solar or geothermal energy supply. To further reduce the number of nodes, not all SNs can communicate with RNs directly.

To overcome the above challenges, this study focuses on the problem of RN deployment with infinite energy for multiple hops between SNs under continuous space. It can be seen that Swarm Intelligence algorithms (SI) have been widely used for these types of problems and indeed provide a better approximate solution to this type of problem. Among them, in recent years, the Artificial Bee Colony algorithm (ABC) [19] with strong global optimization ability and simple structure has received much attention. It has been widely used in the combinatorial optimization problem [20], complex weight matrix derivative problem [21], and other problems. In the literature, Hashim et al. [16] used this algorithm for the optimization of WSNs. However, in previous studies the characteristics of the problem have not been deeply integrated with the algorithm, and the algorithm needs a lot of adjustment parameters to adapt to the characteristics of the problem; additionally, the parameters of the SI algorithm are often relatively large, and it is difficult to obtain a balance between the global and local search ability of the algorithm. This paper proposes an improved Artificial Bee Colony algorithm based on problem characteristics and dynamic search balance, then uses this algorithm to solve deployment problems and compares it with other algorithms. Simulation experiment results verify that the proposed algorithm improves the global search capability, result stability, and local convergence speed for different problems. This study mainly contributes to optimizing lifetime by RN deployment in TT-WSN, in particular as follows:

- The dimensional characteristics of the problem are incorporated into the search formula of the ABC. This enables the algorithm to adjust the search step according to the dimensionality of the problem, improving the global search capability of the algorithm in a targeted manner.
- The adaptation degree of each individual in the operation of the population intelligence algorithm is integrated into the search process of the algorithm. This helps the algorithm to adjust the speed of local convergence according to the adaptation degree and strengthens its local convergence ability.
- The dynamic search balance strategy is used to replace the scout bee phase in traditional ABC to further reduce the algorithm parameters and improve the ease of using the algorithm.
- Based on the proposed two-layer WSN backbone network algorithm, the problem of deploying different numbers of RNs under different scenarios is studied and a general deployment method for lifetime optimization is obtained.

The rest of this paper is organized as follows. Section 2 models the problem for TT-WSN. In Section 3, we first provide a general overview of the base ABC algorithm, then describe the proposed algorithm and how to use the proposed method in TT-WSN in detail. Finally, the proposed algorithm is applied to solve the problems of TT-WSN relay node deployment in Section 4, and the paper is concluded in Section 5.

## 2. Relay Node Deployment Problem for TT-WSN

### 2.1. Network Model

**Figure 1:**
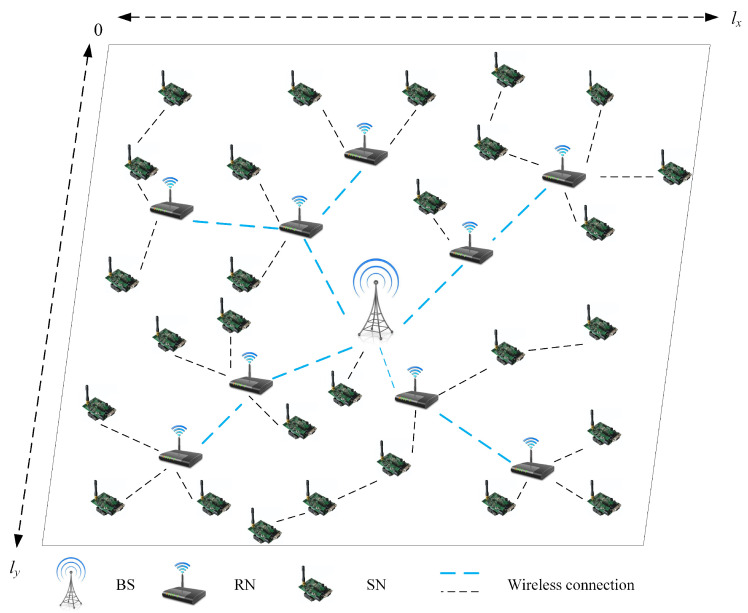
TT-WSN illustration.

This research studies a TT-WSN with size $l_{x}\times l_{y}$. The equipment deployed includes $N_{s}$ SNs, $N_{r}$ RNs, and one sink node (BS), as shown in Figure 1. All SNs have the same initial energy, i.e., RNs only forward without collecting information and it is assumed that their energy is unlimited. SNs and RNs follow the unit disk communication model. The condition for any two devices to communicate with each other is that their Euclidean distance be less than or equal to the communication distance. SNs can send information to SNs, RNs or the BS, while RNs can only send information to other RNs or the BS. The routing protocol is provided by the shortest energy consumption path calculated by the SPFA algorithm [22]. The medium access protocol uses the S-MAC protocol [23]. All SNs collect data and send it to the BS. This process is a work cycle; for simplicity, it is assumed that each work cycle takes 10 min.

### 2.2. Energy Consumption Model

The energy model used in this study mainly considers the most energy-intensive data transmission process while ignoring the energy consumption of the process of reception, processing, and perception [24].

At time t>0(t∈τ), the data packet $P_{i}\left(t\right)$ sent by the sensor *i* includes the data packet forwarded by the sensor and the data packet generated by the sensor through perception. The sensor generates a data packet at every time unit.

(1) $$P_{i}\left(t\right)=1+\sum_{j\in \left\{S_{s}\left(t\right)-i\right\}}\kappa_{j,i}^{s}\left(t\right)\;\;t>0$$

Here, $S_{s}\left(t\right)$ is the set of sensor coordinates with remaining power greater than 0, and $S_{s}\left(t\right)\subseteq S_{s}$, $S_{s}$ is the set of all sensor coordinates at the beginning. When time t>0 and $i\in S_{s}\left(t\right)$ is in the shortest path between $j\in S_{s}\left(t\right)$ and the receiving node, $\kappa_{j,i}^{s}\left(t\right)$ is equal to 1.

When i>0, the amount of energy consumed by the sensor *i* is provided as the equation below:

(2) $$EP_{i}\left(t\right)=P_{i}\left(t\right)\cdot amp\cdot k\cdot ||i-\varsigma_{i}^{s}\left(t\right)|{|}^{\alpha }\cdot \beta .$$

Here, amp is the energy consumed by the power amplifier per bit (amp>0), *k* is the size of the data packet in bits, ||·|| is the Euclidean distance between the two devices, $\varsigma_{i}^{s}\left(t\right)$ is the next device on the shortest path, α is the communication attenuation index (α∈[2,4]), and β is the transmission quality parameter (β>0). This equation simulates the additional energy loss due to packet loss.

The initial energy possessed by the sensor is ie. At time *t*, the remaining energy of the sensor *i*, $EL_{i}\left(t\right)$ is expressed as follows:

(3) $$EL_{i}\left(t\right)=\left\{\begin{array}{c} EL_{i}\left(t-1\right)-EP_{i}\left(t\right)\;\;\;t>0\\ ie\;\;\;\;\;\;\;\;\;\;\;\;\;\;\;\;\;\;\;\;\;\;\;\;t=0 \end{array}\right.$$

### 2.3. Lifetime Definition

Network lifetime is defined in this study as the number of time periods in which WSN can provide valid information, as shown in Equation (4). When any sensor runs out of energy, the network is considered to have reached the maximum life limit [25]:

(4) $$lt=|\left\{t>0\in \tau ,\exists EL_{i}=0\right\}|\;\;i\in S_{s}$$

where lt is the lifetime of the network when at least one sensor runs out of energy.

The main goal of this research is to study how to maximize lt by reasonably deploying relay nodes in TT-WSN.

## 3. Implementation of pdABC for TT-WSN Relay Node Deployment

### 3.1. Overview of ABC

Inspired by the foraging process of honey bee swarms, Karaboga [19] proposed the ABC to simulate similar processes. The algorithm is mainly used to solve nonlinear optimization problems with multiple peaks and valleys in multiple dimensions. The algorithm consists of four main phases.

In the beginning, Equation (5) is used to generate random solutions as the initial food sources:

(5) $$x_{ij}=x_{j}^{\min}+\lambda \left(x_{j}^{\max}-x_{j}^{\min}\right)$$

where i=1,2,⋯,SN; j=1,2,⋯,D; $x_{j}^{\min}$ and $x_{j}^{\max}$ are the lower and upper bounds for the index *j*, respectively; SN stands for half of the colony size; *D* is the dimension of the problem; and λ is a random real number within the range [0, 1].

The following Equation (6) is applied to the generated food sources to calculate their fitness. It is used to indicate the goodness of the solution, and the best food source is memorized.

(6) $$fit_{i}=\left\{\begin{array}{c} \frac{1}{1+f_{i}},\;\;\;\;\;\;\;\;\;f_{i}\geq 0\\ 1+|\left(f_{i}\right)|,\;\;\;f_{i}<0 \end{array}\right.$$

where $fit_{i}$ represents the fitness of solution *i*, $f_{i}$ is the result of objective function, and i∈{1,2,⋯,SN}.

Then, the employed bees begin to explore the food sources. Each employed bee uses Equation (7) to generate a candidate food source, i.e., a candidate solution:

(7) $$v_{ij}=x_{ij}+\phi_{ij}\left(x_{ij}-x_{kj}\right)$$

where *j* is a randomly selected dimension such that j∈{1,2,⋯,D}, *k* is a randomly chosen food source such that k∈{1,2,⋯,SN}, and k≠i. $\phi_{ij}$ is produced randomly in the range [−1, 1]. If the candidate solution is better than the original solution, the candidate solution replaces the original solution; conversely, the original solution remains unchanged, and the number of times the food source is not updated increases by one.

Further, before the onlooker bees explore food sources, the probability *p* of each food source being selected is first calculated using the following Equation (8) [26]

(8) $$p_{i}=0.9\times \frac{fit_{i}}{\max(fit_{i})}+0.1$$

The onlooker bees select the food sources based on the probability. Then, they explore the food source according to Equation (7) and generate new candidate food sources. They determine whether the current food source is updated. If it is not updated, the number of times the food source remains unchanged increases.

When the number of times a food source is not updated exceeds a set value, an employed bee turns into a scout bee using Equation (5) for food source refreshment. This allows the algorithm to jump out of local optimal solutions.

In the above process, the respective number of employed and onlooker bees is equal to half of the population size, meaning that there is only one scout bee.

### 3.2. Introduction of pdABC Algorithm

#### 3.2.1. Search Equation Based on Problem Dimension and Fitness

When using Equation (7) in the ABC algorithm, $x_{i}$ is randomly selected. This results in ABC having strong global search capability, poor local search capability, and slow convergence speed [27]. To solve this problem, a new Artificial Bee Colony algorithm, GABC, has been proposed in the literature [27]. The search equation of the algorithm is provided below:

(9) $$v_{ij}=x_{ij}+\phi_{ij}\left(x_{ij}-x_{kj}\right)+\varphi_{ij}\left(y_{j}-x_{ij}\right)$$

where $\varphi_{ij}$ is a random number between [0, C], C is a non-negative constant, and $y_{j}$ is the *j*th component of the global optimal solution. The search equation enhances the local search ability of the algorithm to an extent without affecting its global search ability.

According to the general optimization process, the algorithm requires a strong global search ability in the early stage of optimization to ensure that the region where the optimal solution is located can be detected, as well as a strong local search ability in the later stagein order to converge quickly. It can be seen from Equation (9) that the randomness of $\varphi_{ij}$ further improves the convergence speed of the GABC algorithm [28]. In addition, in the process of testing other ABC variants [28,29,30], it is often apparent that different optimization strategies only perform better on some benchmark functions. Therefore, it is necessary to redesign the search equation to ensure that it can better balance the exploration and development capabilities of the algorithm, thereby improving its adaptability to different problems. To achieve the above purpose, we propose the pdABC algorithm for the following search equation:

(10) $$v_{ij}=x_{ij}+\phi_{ij}\cdot f_{g}\cdot \left(x_{ij}-x_{kj}\right)+C\cdot f_{best}^{l}\cdot \left(y_{j}-x_{ij}\right)$$

where $f_{g}$ is a variable used to balance the global search and local search of the algorithm, and is defined specifically in the equation below:

(11) $$f_{g}={\left(1-l\right)}^{1/D}$$

where *D* is the demension of the problem and *l* is a linearly varying parameter, which is defined as follows:

(12) l=Curitr/Maxitr

where Maxitr is the maximum number of iterations of the algorithm and Curitr is the number of iterations where the algorithm is currently located.

The part $f_{best}^{l}$ is used to adjust the search speed of the algorithm elastically in order to adapt the algorithm to different problems, as expressed in the equation below:

(13) $$f_{best}^{l}={\left(1+e^{-(fitness_{i}/fitness_{\min})}\right)}^{l}$$

where $fitness_{i}$ is the fitness of individual *i* and $fitness_{\min}$ is the minimal fitness. It can be noticed that *l* belongs to [0, 1] due to Equation (12) gradually approaching 1 as the iterations increase, while $f_{best}^{l}$ gradually becomes bigger. This means that the second part in Equation (10) has an increasing impact with increasing iterations. The exploitation ability of the algorithm is gradually increased, speeding up the convergence of the algorithm. In addition, the introduction of the fitness values serves to adjust for different problems. It is worth noting that the fitness value in this work refers to the network lifetime. We introduce $fitness_{\min}$, which means the current best lifetime. Through the ratio of $fitness_{i}$ to $fitness_{\min}$, the value in the $f_{best}^{l}$ is greatly reduced to meet the needs of practical purposes. In summary, we are able to find that with this part of the equation the algorithm tends to converge faster when the number of iterations increases or the lifetime acquired by a particular bee is larger.

It can be seen from Equation (10) that increased *l* leads to the decrease of $f_{g}$; the increase of $f_{best}^{l}$ means that the algorithm has a strong global search ability in its early stage, which is attenuated in the later stage, and its local search ability is enhanced to accelerate its convergence. Here, *D* is the dimension of the problem. When *D* is large, it means that the problem is complex; at this time, it means that the algorithm has strong global optimization ability to meet the needs of complex problems. When *D* is small, the opposite is true. The introduction of the adaptation value in Equation (13) enables $f_{best}^{l}$ to adjust the search speed according to different problems, thereby enhancing the adaptability of the algorithm to different problems.

#### 3.2.2. Dynamic Search Balance Strategy

**Figure 2:**
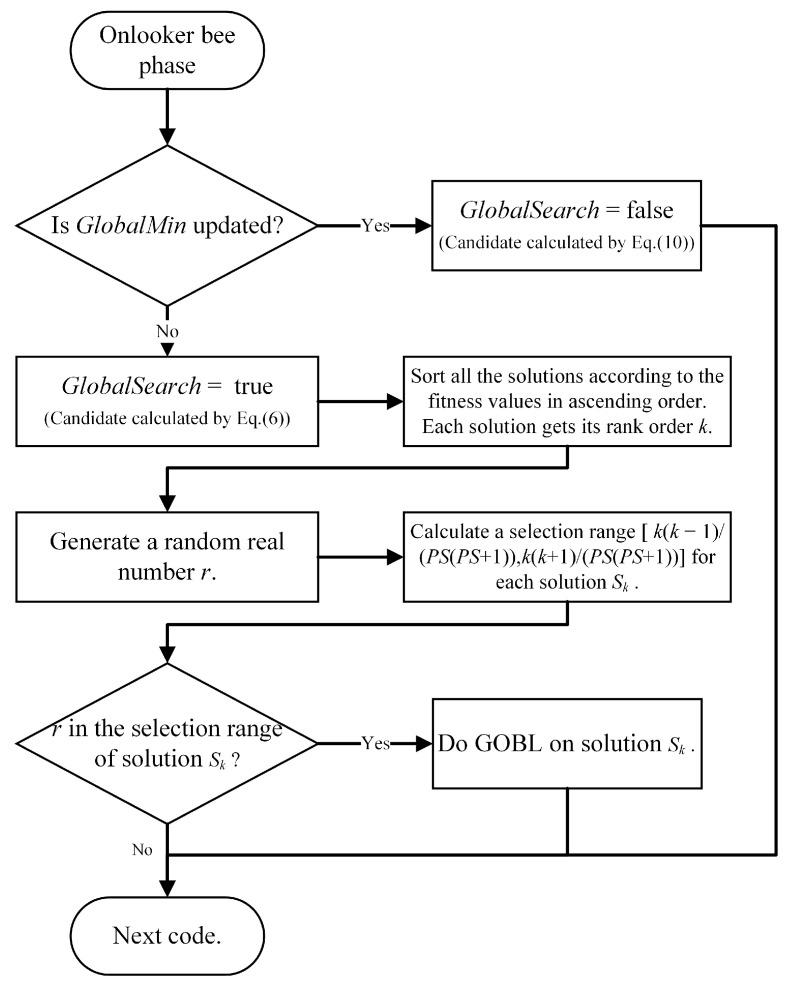
Flow Chart of Dynamic Search Update Method.

In order to accelerate the convergence speed of the algorithm and obtain a more accurate solution, in Equation (10), its global optimization ability is attenuated according to a certain rule, and its local optimal ability is gradually enhanced. However, because it is unclear when an individual or several individuals in the algorithm can reach the region where the optimal solution in the solution space is located, the algorithm may fall into a local optimum, although Equation (10) helps to balance the global and local optimization ability of the algorithm. In order to solve this problem, we propose a dynamic search update strategy to balance the global optimization and local optimization abilities of the algorithm. We use this strategy to replace the reconnaissance stage of the traditional Artificial Bee Colony algorithm. The specific process of our dynamic search balancing strategy is described in the flow chart of Figure 2. The main idea behind GOBL [30] is that when evaluating a candidate solution *S* for a given problem, the solution $\bar{S}$ at the opposite position is calculated at the same time. $\bar{S}$ has a higher probability of approaching the optimal solution of the problem than the randomly selected solution. In this algorithm, the dynamic search balancing strategy replaces the detection bee stage in ABC, meaning that the parameters in ABC no longer need to be considered.

### 3.3. The Proposed Algorithm

**Figure 3:**
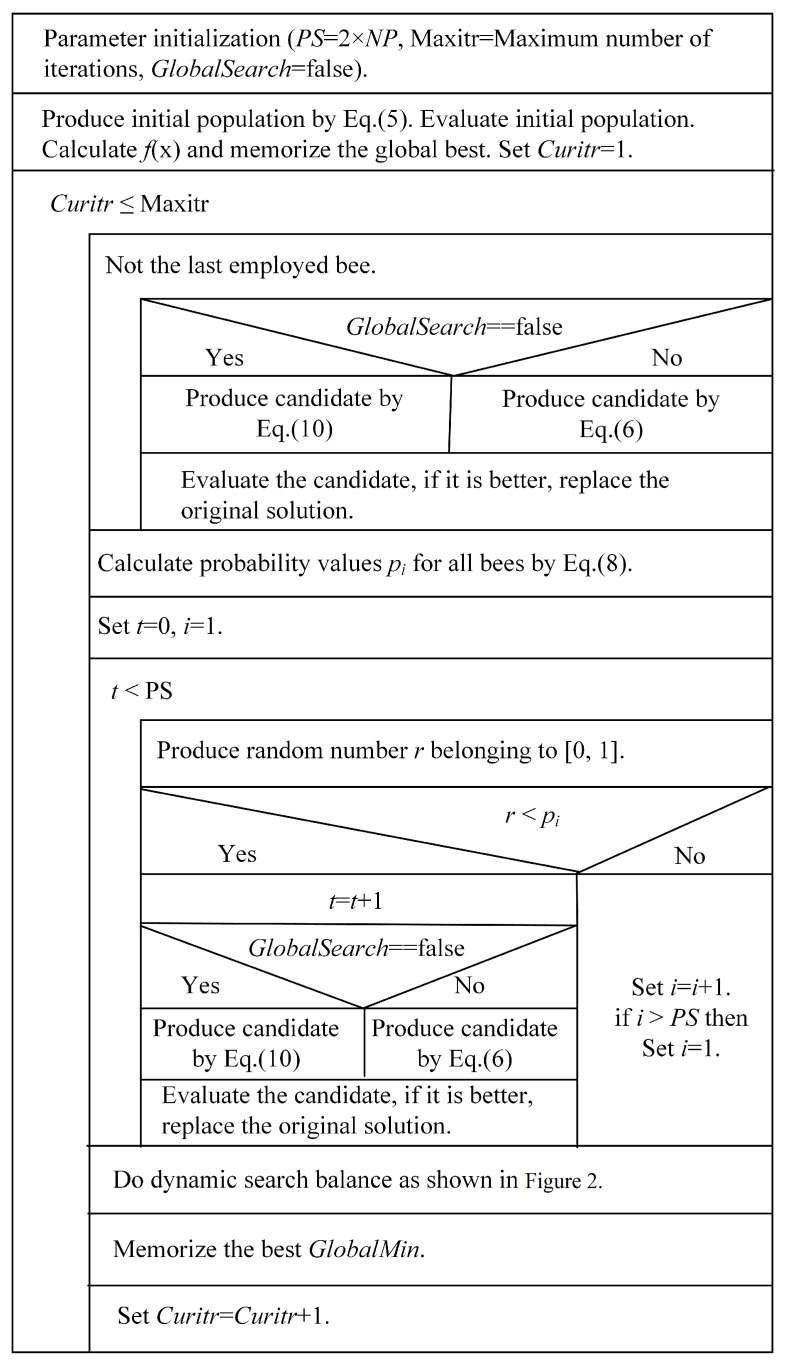
N-S Diagram of dpABC.

Compared with the original ABC algorithm and several other ABC variants, dpABC algorithm differs in three areas: first, a new search equation with dynamically changing global and local search capabilities is used in the employed and onlooker bee phases; second, a dynamic search balancing strategy is used to fully prevent the algorithm from falling into local optima; and third, the scout bee phase is replaced by dynamic search balancing, meaning that the parameter limit in the original algorithm does not have to be considered. The N-S diagram of the dpABC algorithm is shown in Figure 3.

Regarding the complexity of the algorithms, dpABC and ABC have the same complex time in the employed bee, onlooker bee, and probability calculation phases. In the phase of scout bee, due to the dynamic search balance strategy adopted in the dpABC algorithm, the merging sort used in this method results in higher time complexity O(NlogN) than that of ABC O(N). However, this complexity can be controlled by limiting the number of problem calculations. Moreover, although the dpABC algorithm has a relatively high time complexity, its global optimization ability and local convergence acceleration capability are enhanced, as confirmed in the simulation section.

### 3.4. Introduction of pdABC into TT-WSN Relay Node Deployment

#### 3.4.1. Individual Representation, Initialization, and Fitness Value Assignment

**Figure 4:**
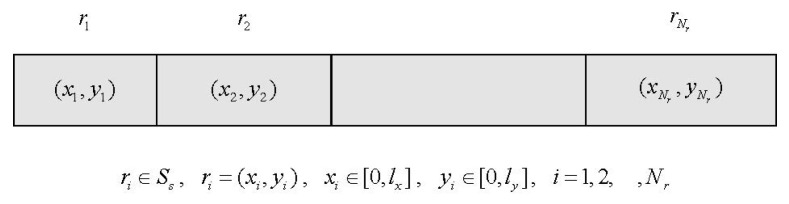
Individual representation.

The individual composition of the algorithm is shown in Figure 4. Each individual consists of the coordinates of $N_{r}$ RNs. During initialization, the coordinates of each RN $r_{i}$ in the individuals are randomly generated according to Equation (14).

(14) $$\left\{\begin{array}{c} r_{i}=\left(x_{i},y_{i}\right)\\ x_{i}=random\left[0,l_{x}\right]\\ y_{i}=random\left[0,l_{y}\right] \end{array}\right.\;\;\;i=1,2,\cdots N_{r}$$

In this approach, network lifetime is used as fitness.

(15) $$fitness_{i}=lt_{i}$$

where *i* represents the *i*th individual in the population.

#### 3.4.2. Feasible Solution Formation

The WSN used in this study has two layers, with the relay nodes forming the upper layer backbone network. Such a set of relay node coordinates is called a feasible solution of the network. In the process of randomly deploying nodes, due to the limited communication distance of relay nodes, a certain relay node may be too far away from other relay nodes to communicate with them. To solve this problem, when the coordinates of a relay node are determined, a feasible solution of the algorithm can be formed. The following steps are specifically used to form the backbone network of the relay node: Step 1. $S_{r}=\left\{All\;relay\;nodes\right\}$, S={Thesinknode}; Step 2. Find all nodes in $S_{r}$ that can communicate with at least one node in *S* to form a set ${S_{r}}^{\prime }$; Step 3. If ${S_{r}}^{\prime }$ is empty, find the two closest points $r_{2}$ and $r_{1}$ in $S_{r}$ and *S*, respectively; $r_{2}$ moves toward $r_{1}$ straightly until the distance between them is equal to or less than $r_{c}$. If there are two or more nodes in $S_{r}$, which are at the same distance with respect to a node in *S*, choose any one of them; Step 4. ${S_{r}}^{\prime }={S_{r}}^{\prime }\cup \left\{r_{2}\right\}$; Step 5. $S=S\cup {S_{r}}^{\prime }$; Step 6. $S_{r}=S_{r}-S$; Step 7. If $S_{r}$ is empty, the iteration stops; otherwise, go to step 2. Through the above virtual movement process of the relay nodes, the formation of the TT-WSN backbone network is ensured.

## 4. Numerical Experiments

Based on the above research, the TT-WSN network lifetime can be optimized. In this section, we describe the different RN deployment experiments we conducted. First, the scenarios of a specific TT-WSN deployment are provided, including the source of the data reference. Then, the network parameters of the TT-WSNs are depicted, along with the specific parameters of the algorithms. Numerical simulations are run for different scenarios and different number of RNs to analyze and study the global optimum, convergence speed, and robustness of the algorithm in multiple cases. In order to further verify the performance of the proposed algorithm, a number of more widely used algorithms from recent years were selected for comparative analysis and research.

### 4.1. Configuration of Network Parameters

#### 4.1.1. Experimentsal Scenario

**Figure 5:**
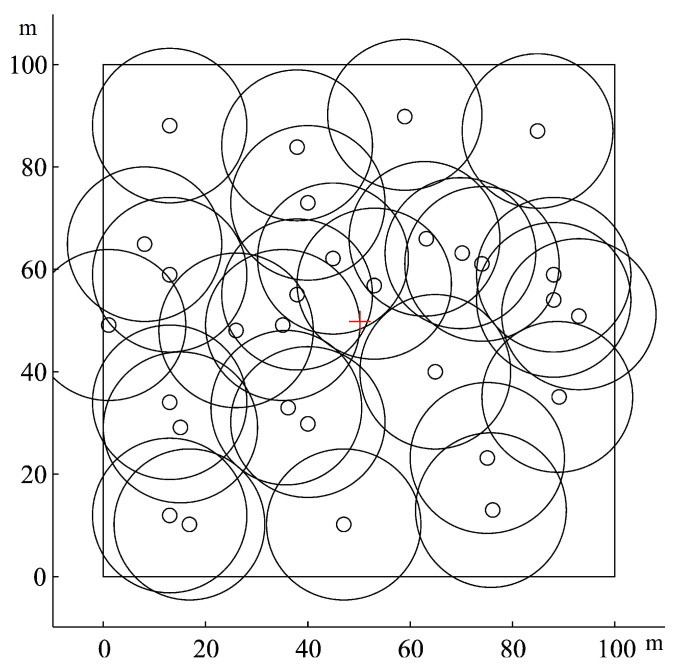
Senario of SN and RN deployment within 100×100 m${}^{2}$.

**Figure 6:**
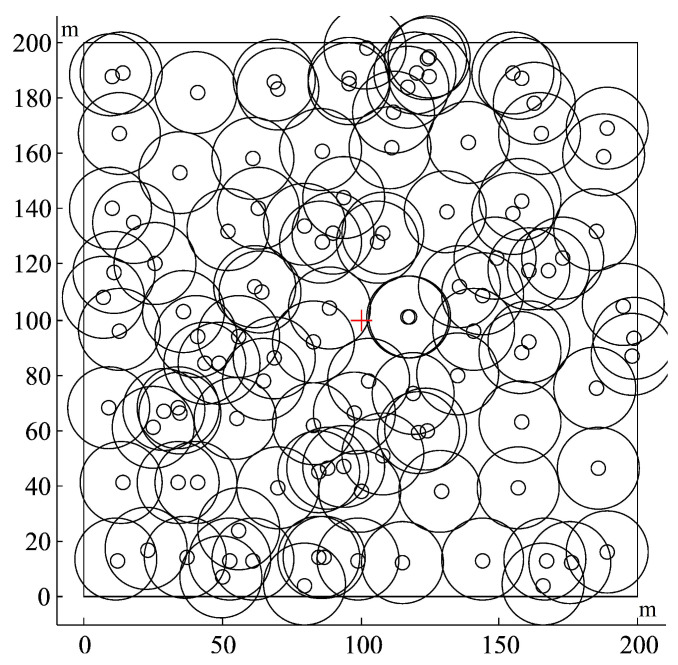
Senerio of SN and RN deployment within 200×200 m${}^{2}$.

The experimental scenarios include 100×100 m${}^{2}$ and 200×200 m${}^{2}$. The numbers of SNs deployed are 30 and 114, respectively. The BS is deployed in the center of the area. Referring to the literature [4], half of the SNs in each scenario are deployed in specific locations and the other half are randomly deployed. The deployment is shown in Figure 5 and Figure 6. The sign “+” represents the BS and the circles represent the SNs in both figures. Due to the high cost of RNs, the number of RNs in this study does not exceed 20% of the number of SNs. The number of RNs that can be deployed in the former scenario varies from 1 to 6, and the number of RNs that can be deployed in the latter scenario varies from 1 to 22.

The experimental program code was written on the JDK 1.7.0_17_64 platform. The specific configuration of the simulation computer running the program was an Intel(R) Xeon(R) CPU E5-2650 v2 2.60 GHz × 2, with 128 GB RAM, and the operating system was Windows Server 2008 R2 Enterprise.

#### 4.1.2. Network Model and Algorithm Configurations

The parameters of the TT-WSN network model were as follows: α=2, β=1, k=128 KB, $r_{s}=15$ m, $r_{c}=30$ m, amp=100 pJ/bit/m${}^{2}$ and ie=10 J [31].

Three other optimization algorithms, namely, Genetic Algorithm (GA) [12], Simulated Annealing Algorithm (SA) [12], and ABC, were employed to check the effectiveness of the pdABC. The population size of all the algorithms was set to 40. The maximum number of iterations for each algorithm was 300. The calculation was repeated 30 times for each instance. The composition of ABC, GA, and SA individuals was the same as that of pdABC. The comparison algorithms all used parameters that needed to be adjusted. For GA, these parameters were the crossover rate and the mutation rate; for SA they were the initial temperature and temperature drop rate; pdABC used the constant parameter C; finally, ABC was set to half the population size multiplied by the dimension of the problem. The parameter selection ranges of the three algorithms that require parameter tuning are shown in Table 1. Each parameter combination was run 30 times with a maximum number of iterations of 100, using 100×100 terrain to deploy six relay nodes. Comparing the final average results, the final selected optimal parameter combinations are listed in the fourth column of Table 1.

### 4.2. Relay Node Deployment Experiments

**Figure 7:**
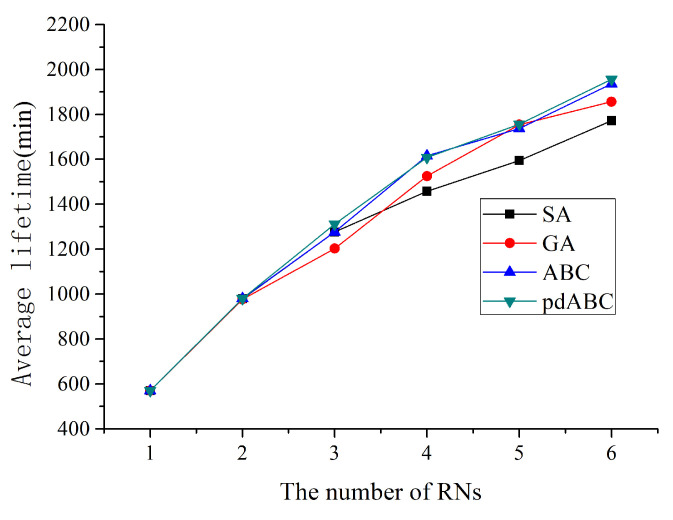
WSN lifetime optimization with different number of RNs in 100×100 terrain.

**Figure 8:**
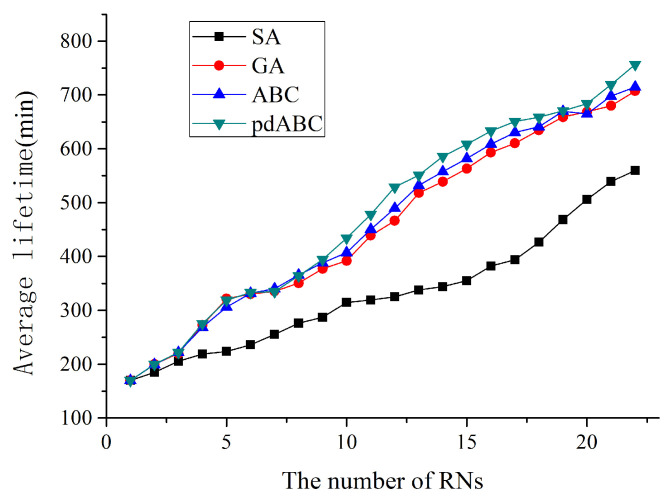
WSN lifetime optimization with different number of RNs in 200×200 terrain.

When the network is only composed of SNs, the lifetime of a network with 30 sensor nodes deployed in 100×100 terrain is 480 min, while the lifetime of a network of 114 sensor routers in 200×200 terrain is 110 min. On this basis, RNs can be added to the network in a gradually increasing manner to observe the network life optimization curve in the 100×100 and 200×200 terrains with different numbers of RNs added, as shown in Figure 7 and Figure 8.

When an RN is added, the optimized lifetime results of 100×100 and 200×200 deployed WSNs are 570 min and 170 min, respectively, 18.75% and 54.54% longer than the WSN without an RN node. This means that adding an RN to an ordinary WSN has a great effect in improving network life. When an RN node is added, all four algorithms compared here obtain the same optimization results. However, as the number of deployed RN nodes continues to increase, the differences between the different algorithms increase. In the 100×100 network, the pdABC algorithm performs slightly better than the ABC algorithm for lifetime optimization; in turn, the ABC algorithm is better than GA algorithm, while the SA algorithm has the worst performance when the number of RNs exceeds three. In the 200×200 terrain, the optimization effect of the different algorithms is more different, and the optimization effect of the pdABC algorithm is further reflected.

**Figure 9:**
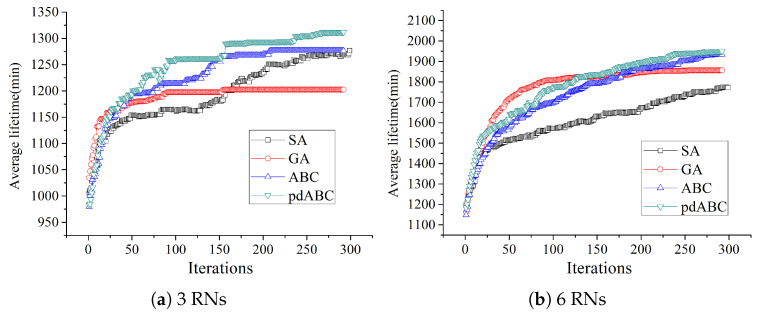
Lifetime optimization trend with different RN numbers in 100×100 terrain.

**Figure 10:**
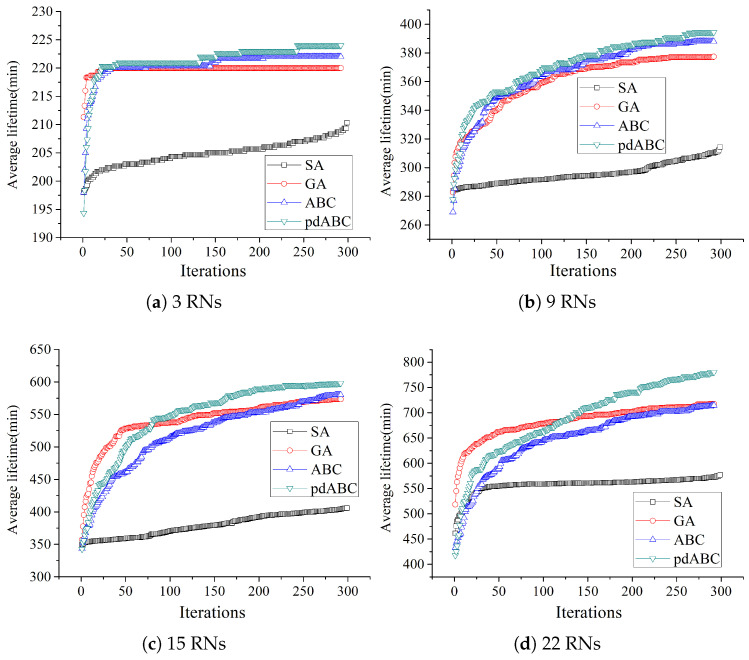
Lifetime optimization trend with different RN numbers in 200×200 terrain.

In order to verify the convergence of the algorithm in solving the TT-WSN relay node deployment problem, the relationship between the different algorithms in terms of lifetime optimization based on the different numbers of RNs and number of iterations is shown in Figure 9 and Figure 10.

When the number of RNs is small, that is, when the problem is low-dimensional, the performance of the algorithms is the same or similar. In order to fully demonstrate the convergence speed of the different algorithms in the different dimensions, the number of RNs is 3 and 6 in the 100 × 100 terrain, and the number of RNs is 3, 9, 15, and 22 in the 200 × 200 terrain. It can be seen from Figure 9 and Figure 10 that the GA algorithm has the fastest convergence speed; however, compared to ABC and pdABC, it can easily fall into a local optimum. The SA algorithm is more likely to fall into local optima in the 200 × 200 terrain than in the 100 × 100 terrain, and it has the worst performance. The convergence speed of pdABC is faster than that of ABC, though weaker than that of GA. As the number of RNs increases and the number of iterations increases, however, pdABC is able to explore better solutions and show stronger global optimization. Compared with the other algorithms, the pdABC algorithm has better ability to avoid falling into local optima.

**Figure 11:**
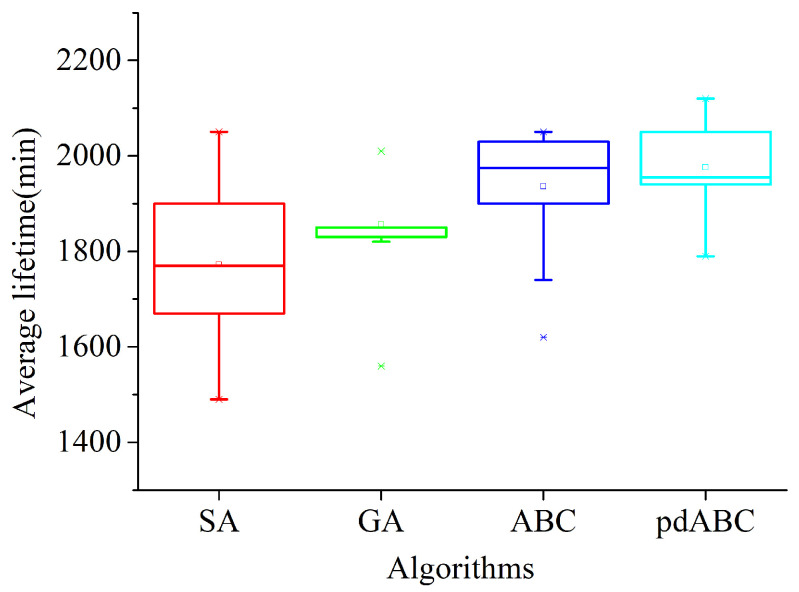
Optimal lifetime distribution box plot with six RNs in 100×100 terrain.

**Figure 12:**
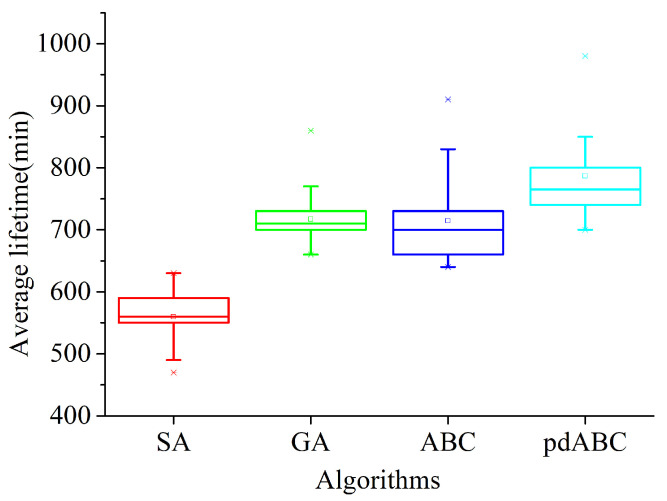
Optimal lifetime distribution box plot with twenty-two RNs in 200×200 terrain.

SA, GA, ABC, and pdABC are all metaheuristic algorithms, which prevents them from falling into a local optima by increasing the random change of the solution. Randomness provides the algorithms with the ability to explore better solutions, although it makes them more volatile. In order to verify the stability of the pdABC algorithm in the process of solving the deployment problem of relay nodes in TT-WSN, the box plots shown in Figure 11 and Figure 12 demonstrate the distribution of the solution.

The box plot evaluates the symmetry and distribution of the solution distribution using five statistical values in the data (the minimum, first quartile, median, third quartile, and maximum), which can be intuitive. The above shows that the solution distribution is stable. The 6 RNs and 22 RNs in the two terrains have the most changes, respectively; thus, they are used to evaluate the stability of the algorithms under the two terrains. It can be seen from Figure 11 and Figure 12 that the distribution of the GA solutions is the most concentrated, that is, its stability is the best. The SA algorithm has the worst performance in the first terrain, although it performs better in the other terrain; in general, the overall health value of the solution is the lowest. The distribution of the solutions of the pdABC algorithm is better than that of ABC algorithm. Although the stability of the pdABC solution distribution is worse than that of GA, the median and average values of the pdABC solutions compared to other algorithms in the two terrains are the largest. In addition, the maximum value found by pdABC is the largest, which means that the algorithm has stronger global optimization performance.

In addition to comparison with the abilities of the SA, GA, and ABC algorithms in solving the problem of deploying RNs in TT-WSN, we compared the dpABC algorithm with other state-of-the-art algorithms, using the same 100 × 100 terrain with 6 RNs and 200 × 200 terrain with 22 RNs for the simulation. The algorithms compared include WOA [32], SCA [33], and GWO [34]. These three algorithms have been widely studied and applied in recent years. Thus, experiments were conducted to verify the performance of dpABC algorithm. A preliminary experimental study aimed at ensuring the fairness of algorithm comparison was conducted to ensure that all the algorithms had the same number of problem evaluations in one iteration. The results can be comprehensively evaluated through iteration numbers and network lifetime. Thus, the parameter of each algorithm’s population size was set. To study the other parameters of these algorithms, see the related references [32,33,34].

The lifetime optimization results of the different algorithms are shown in Table 2, in which Ave.NL represents the average network lifetime of the TT-WSN and Ave.CT denotes the number of iterations when the algorithm reaches the convergence state. Both parameters were obtained by averaging them over several experiments. However, for simplicity, both were taken directly as integers. As can be seen from the table, while WOA has the best coverage ratio, it requires more iterations due to its slow convergence speed. The dpABC algorithm has the second-highest average network lifetime compared to other algorithms, and its average iteration times are reduced nearly by 15% compared with WOA. It can be concluded that the dpABC algorithm not only achieves a higher average network lifetime, it has a faster convergence rate as well.

## 5. Conclusions

In order to solve the problem of how to optimize the deployment of a certain number of RNs in a TT-WSN to increase network life, this paper proposes an improved and enhanced Artificial Bee Colony algorithm (pdABC). In the pdABC algorithm, the search equation is improved in two aspects: first, that the global search part is adjusted according to the dimensions of the problem and the local search part is constructed according to the fitness value; second, in order to prevent the algorithm from premature convergence, a dynamic search balance strategy is proposed. This strategy is used to replace the traditional ABC scout bee stage. Before using the pdABC algorithm to solve the deployment problem of RNs in a TT-WSN, we first constructed an algorithm for using the RNs to form the upper communication backbone of the network. On this basis, the pdABC algorithm is used for optimizing RN deployment in the TT-WSN. To test the role of the pdABC algorithm in the optimization of RN deployment in a TT-WSN, the ABC, GA, and SA algorithms are compared to our proposed pdABC. The experimental results verify that the pdABC algorithm has strong global optimization ability compared to the other three algorithms, and the obtained RN deployment is more uniform. In terms of convergence speed and stability, the pdABC algorithm is better than the ABC and SA algorithms, although it is inferior to GA. In addition, we compare our algorithm against three current state-of-the-art algorithms, and the analysis results show that our algorithm is very competitive.

In our future work, we hope to compare this algorithm with a variety of other state-of-the-art algorithms from multiple perspectives to further validate its performance. In addition, we aim to apply this proposed algorithm to real-world problems and extend it for multi-objective optimization problems.

## Figures and Tables

**Table 1:** Parameter selection of GA, SA, and dpABC.

Algorithms	Parameters	Value Selected	Range
GA	Mutation	0.2	[0.05,0.1,…,0.95]
	Crossover	0.95	[0.05,0.1,…,0.95]
SA	$T_{0}$	4	[1,2,…,20]
	λ	0.85	[0.05,0.1,…,0.95]
dpABC	C	1.5	[0.5,1.0,…,5.0]

**Table 2:** TT-WSN lifetime optimization for different algorithms.

Algorithms	6 RNs (100 × 100)		22 RNs (200 × 200)	
	Ave.NL	Ave.CT	Ave.NL	Ave.CT
WOA	1955	273	783	326
SCA	1824	358	681	378
GWO	1871	312	724	348
dpABC	1934	232	777	280

## Data Availability

Not applicable.

## References

[1] Amutha J., Sharma S., Nagar J. (2020). WSN Strategies Based on Sensors, Deployment, Sensing Models, Coverage and Energy Efficiency: Review, Approaches and Open Issues. Wirel. Pers. Commun..

[2] Dinh N.T., Kim Y. (2019). Auto-configuration in wireless sensor networks: A review. Sensors.

[3] Sefati S., Abdi M., Ghaffari A. (2021). Cluster-based data transmission scheme in wireless sensor networks using black hole and ant colony algorithms. Int. J. Commun. Syst..

[4] Lanza-Gutierrez J.M., Gomez-Pulido J.A. (2016). Studying the multiobjective variable neighbourhood search algorithm when solving the relay node placement problem in Wireless Sensor Networks. Soft Comput..

[5] Zhou C., Mazumder A., Das A., Basu K., Matin-Moghaddam N., Mehrani S., Sen A. Relay node placement under budget constraint. Proceedings of the 19th International Conference on Distributed Computing and Networking.

[6] Cheng X.Z., Narahari B., Simha R., Cheng M.X., Liu D. (2003). Strong minimum energy topology in wireless sensor networks: NP-completeness and heuristics. IEEE Trans. Mob. Comput..

[7] Misra S., Majd N.E., Huang H. Constrained relay node placement in energy harvesting wireless sensor networks. Proceedings of the IEEE 8th International Conference on Mobile Adhoc and Sensor Systems (MASS).

[8] Misra S., Majd N.E., Huang H. (2014). Approximation Algorithms for Constrained Relay Node Placement in Energy Harvesting Wireless Sensor Networks. IEEE Trans. Comput..

[9] Perez A.J., Labrador M., Wightman P.M. A multiobjective approach to the relay placement problem in wsns. Proceedings of the of Wireless Communications and Networking Conference (WCNC).

[10] Nigam A., Agarwal Y.K. (2014). Optimal relay node placement in delay constrained wireless sensor network design. Eur. J. Oper. Res..

[11] Truong T.T., Brown K.N., Sreenan C.J. (2015). Multi-objective hierarchical algorithms for restoring Wireless Sensor Network connectivity in known environments. Ad Hoc Netw..

[12] Ozkan O., Ermis M. (2015). Nature-inspired relay node placement heuristics for wireless sensor networks. J. Intell. Fuzzy Syst..

[13] Peiravi A., Mashhadi H.R., Javadi S.H. (2013). An optimal energy-efficient clustering method in wireless sensor networks using multi-objective genetic algorithm. Int. J. Commun. Syst..

[14] Azharuddin M., Jana P.K. A GA-based approach for fault tolerant relay node placement in wireless sensor networks. Proceedings of the Third International Conference on Computer, Communication, Control and Information Technology (C3IT).

[15] Chen C.C., Chang C.Y., Chen P.Y. (2015). Linear Time Approximation Algorithms for the Relay Node Placement Problem in Wireless Sensor Networks with Hexagon Tessellation. J. Sens..

[16] Hashim H.A., Ayinde B.O., Abido M.A. (2016). Optimal placement of relay nodes in wireless sensor network using Artificial Bee Colony algorithm. J. Netw. Comput. Appl..

[17] Yang D., Misra S., Fang X., Xue G., Zhang J. (2012). Two-Tiered Constrained Relay Node Placement in Wireless Sensor Networks: Computational Complexity and Efficient Approximations. IEEE Trans. Mob. Comput..

[18] Ma C., Liang W., Zheng M. (2017). PSH: A Pruning and Substitution Based Heuristic Algorithm for Relay Node Placement in Two-Tiered Wireless Sensor Networks. Wirel. Pers. Commun..

[19] Karaboga D. (2005). An Idea Based on Honey Bee Swarm for Numerical Optimization.

[20] Kaya E., Gorkemli B., Akay B., Karaboga D. (2022). A review on the studies employing Artificial Bee Colony algorithm to solve combinatorial optimization problems. Eng. Appl. Artif. Intell..

[21] Wang L., Han S. The improved Artificial Bee Colony algorithm for mixed additive and multiplicative random error model and the bootstrap method for its precision estimation. Geod. Geodyn..

[22] Fanding D. (1994). A Faster Algorithm for Shortest-Ptath–SPFA. J. Southwest Jiaotong Univ..

[23] Ye W., Heidemann J., Estrin D. An energy-efficient MAC protocol for wireless sensor networks. Proceedings of the Twenty-First Annual Joint Conference of the IEEE Computer and Communications Societies.

[24] Konstantinidis A., Yang K., Zhang Q. An evolutionary algorithm to a multi-objective deployment and power assignment problem in wireless sensor networks. Proceedings of the IEEE Global Telecommunications Conference.

[25] Tsai C.-W., Hong T.-P., Shiu G.-N. (2016). Metaheuristics for the Lifetime of WSN: A Review. IEEE Sens. J..

[26] Jadon S.S., Bansal J.C., Tiwari R., Sharma H. (2018). Artificial Bee Colony algorithm with global and local neighborhoods. Int. J. Syst. Assur. Eng. Manag..

[27] Zhu G., Kwong S. (2010). Gbest-guided Artificial Bee Colony algorithm for numerical function optimization. Appl. Math. Comput..

[28] Gao W.F., Liu S.Y., Huang L.L. (2013). A Novel Artificial Bee Colony Algorithm Based on Modified Search Equation and Orthogonal Learning. IEEE Trans. Cybern..

[29] Jadon S.S., Bansal J.C., Tiwari R., Sharma H. (2015). Accelerating Artificial Bee Colony algorithm with adaptive local search. Memetic Comput..

[30] Zhou X.Y., Wu Z.J., Wang H., Rahnamayan S. (2016). Gaussian bare-bones Artificial Bee Colony algorithm. Soft Comput..

[31] Konstantinidis A., Yang K. (2011). Multi-objective k-connected deployment and power assignment in wsns using a problem-specific constrained evolutionary algorithm based on decomposition. Comput. Commun..

[32] Mirjalili S., Lewis A. (2016). The Whale Optimization Algorithm. Adv. Eng. Softw..

[33] Mirjalili S. (2016). SCA: A Sine Cosine Algorithm for solving optimization problems. Knowl.-Based Syst..

[34] Mirjalili S., Mirjalili S.M., Lewis A. (2014). Grey Wolf Optimizer. Adv. Eng. Softw..

